# Diagnostik von Beinödemen

**DOI:** 10.1007/s00105-022-05082-6

**Published:** 2022-12-09

**Authors:** Markus Stücker, Kerstin Protz, Stefan Eder, Severin Läuchli, Jürg Traber, Joachim Dissemond

**Affiliations:** 1 Klinik für Dermatologie, Venerologie und Allergologie, Venenzentrum der Dermatologischen und Gefäßchirurgischen Kliniken, Kliniken der Ruhr-Universität Bochum, Im St. Maria-Hilf-Krankenhaus, Bochum, Deutschland; 2 Competenzzentrum Versorgungsforschung in der Dermatologie (CVderm), Institut für Versorgungsforschung in der Dermatologie und bei Pflegeberufen (IVDP), Universitätsklinikum Hamburg-Eppendorf (UKE), Hamburg, Deutschland; 3 Schwarzwald-Baar Klinikum, Klinik für Gefäßchirurgie und Gefäßmedizin, Villingen-Schwenningen, Deutschland; 4 Dermatologisches Zentrum Zürich AG, Zürich, Schweiz; 5 Venenklinik Bellevue Kreuzlingen (VBK), Kreuzlingen, Schweiz; 6 Klinik und Poliklinik für Dermatologie, Venerologie und Allergologie, Universitätsklinikum Essen, Hufelandstr. 55, 45122 Essen, Deutschland

**Keywords:** Lymphödeme, Lipödeme, Godet-Zeichen, Sonographie, Wasserplethysmographie, Lymph edema, Lipedema, Godet’s sign, Sonography, Water plethysmography

## Abstract

Ödeme der unteren Extremitäten entsprechen immer einem pathologischen Zustand, der insbesondere bei Betroffenen mit chronischen Wunden einer Therapie bedarf. Weil die Ursachen dieser Ödeme sehr unterschiedlich und teilweise auch komplex sein können, sollte zuerst eine klinische und ggf. apparative Diagnostik erfolgen. Oft kann nach einer klinischen Untersuchung mit Testung des Stemmer- und Godet-Zeichens bereits eine klinische Verdachtsdiagnose gestellt werden. Als weiterführende apparative Diagnostik kann eine sonographische Untersuchung erfolgen. Messtechniken wie beispielsweise die Wasserplethysmographie gelten derzeit zwar als Goldstandard für Volumenmessungen, sind aber sehr aufwendig und fehleranfällig, sodass sie in der klinischen Routine heute kaum angewendet werden. Zusammenfassend wird empfohlen, für die Ödemdiagnostik eine klinische Untersuchung möglichst in Kombination mit einer Sonographie durchzuführen. Insbesondere zu Beginn der Entstauungsphase sollten regelmäßig Umfangsmessungen durchgeführt und dokumentiert werden. Diese Dokumentation ist für die Bewertung des therapeutischen Erfolgs von hoher Aussagekraft.

## Abstract (en)

Edema of the lower extremities is always associated with a pathological condition that should be treated, especially in patients with chronic wounds. Because the underlying causes of edema can vary greatly and sometimes be complex, clinical and, if necessary, various diagnostic tests should also be performed. Often, a suspected clinical diagnosis can already be made after clinical inspection with testing of Stemmer’s and Godet’s signs. Sonographic examination should then be performed as the next diagnostic test. Although measurement techniques such as water plethysmography are currently considered gold standard for volume measurements, they are very complex and prone to error, so that they are rarely used in clinical routine today. In summary, it is recommended to perform a clinical examination, if possible in combination with sonography, for edema diagnosis. Especially at the beginning of the decongestion phase, regular circumferential measurements should be performed and documented. This documentation is of high relevance for evaluation of therapeutic success.

Als Ödeme bezeichnet man Schwellungen von Körpergewebe, die durch eine Ansammlung von Flüssigkeiten im interstitiellen Raum entstehen [14]. Eine Ödembildung ist immer ein pathologischer Zustand, der unter anderem die Mikrozirkulation sowie die Regeneration von Gewebe behindert und somit beispielsweise die Wundheilung erschwert [19]. Die Ausbildung von Ödemen kann verschiedene Ursachen haben (Tab. 1).

**Table 1:** 

Adipositas-assoziierte Ödeme
Allergische Ödeme
Eiweißmangelödeme
Hepatische Ödeme
Kardiale Ödeme
Lipödeme
Lymphödeme
Medikamenteninduzierte Ödeme
Ödeme durch Infektionskrankheiten
Orthostatische Ödeme
Phlebologische Ödeme
Posttraumatische Ödeme
Renale Ödeme
Schwangerschaftsödeme

Die Einschränkungen der Betroffenen durch das Auftreten von Ödemen können sehr vielfältig sein [12]. Wenn Ödeme an den unteren Extremitäten auftreten, beschreiben Betroffene oft ein Schwere- und Spannungsgefühl bis hin zu Schmerzen. Die Beweglichkeit und die Fähigkeit, den Alltag selbstständig zu gestalten, nehmen ab, und die Lebensqualität der Betroffenen ist erheblich beeinträchtigt. Zudem kann es zu Hautveränderungen, wie beispielsweise Stauungsekzemen mit Pruritus und Blasen, kommen. Daher sollten Ödeme, unabhängig von deren Genese, im Rahmen der Wundbehandlung immer adäquat diagnostiziert und therapiert werden.

In diesem Übersichtsbeitrag sollen die aktuellen Möglichkeiten der Diagnostik von Beinödemen insbesondere mit dem Fokus auf die Betroffenen mit chronischen Wunden der unteren Extremitäten dargestellt werden.

## Differenzialdiagnose von Ödemen

Die Diagnose der Ursache(n) von Ödemen ist für die weitere Therapie von größter Bedeutung. Hier sind Anamnese, klinische Untersuchung und auch apparative Untersuchungsverfahren erforderlich, um außerhalb der Extremität gelegene Ursachen wie beispielsweise Herzinsuffizienz, renale oder hepatische Erkrankungen auszuschließen. Eine apparative Diagnostik ist ebenfalls erforderlich, um durch insuffiziente Venen bedingte Ödeme nachzuweisen bzw. auszuschließen. Für die Differenzierung zwischen Phlebödemen, Lymphödemen und Lipödemen sind zudem das Verteilungsmuster sowie das Vorhandensein des Stemmer- und des Godet-Zeichens hilfreich (Tab. 2).

**Table 2:** 

	Phlebödem	Lymphödem	Lipödem
Fuß betroffen	Ja	Ja	Nein
Zehen betroffen	Nein	Ja	Nein
Stemmer-Zeichen	Negativ	Positiv	Negativ
Godet-Zeichen	Positiv	Negativ	Negativ

## Klinische Untersuchung

Nach der Anamnese sollte immer eine klinische Untersuchung der Patienten erfolgen. Grundsätzlich wäre hier ein Ganzkörperstatus wünschenswert. Im Kontext der ambulanten Wundbehandlung von Menschen mit chronischen Wunden an den unteren Extremitäten sollte aber zumindest eine klinische Untersuchung beider Unterschenkel und Füße erfolgen. Es wird dabei u. a. untersucht, ob ein Ödem vorliegt, ob es seitengleich verteilt ist, wie sich dieses tastet und welche Regionen betroffen sind. Aufgrund dieser Resultate ergeben sich dann bereits erste Hinweise auf die Genese der Ödeme.

### Lymphödeme

**Figure 1:**
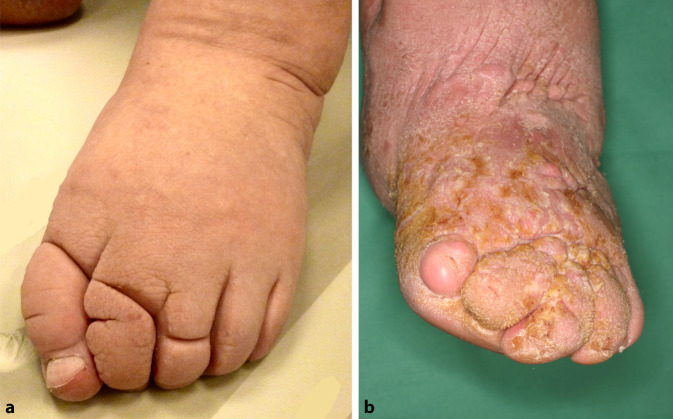

Auch heute werden für die teils massiven Schwellungen bei Lymphödemen oft noch die Begriffe Elephantiasis (nostras) oder Elefantenbein verwendet. Da dies von Betroffenen und Angehörigen als negativ und stigmatisierend empfunden werden kann, empfiehlt die Initiative Chronische Wunden (ICW) e. V., diese Begriffe nicht mehr zu verwenden, sondern stattdessen die Einteilung entsprechend der aktuellen AWMF(Arbeitsgemeinschaft der Wissenschaftlichen Medizinischen Fachgesellschaften e. V.)-Leitlinie zu nutzen [7]. Lymphödeme können entsprechend der aktuellen AWMF-Leitlinie Diagnostik und Therapie der Lymphödeme [2] in verschiedene Stadien eingeteilt werden (Tab. 3). Weitere typische klinische Zeichen des Lymphödems sind die braun-grau verfärbte Haut, Pachydermie, tiefe Querfalten über den Zehengrundgelenken, Ausbildung von Kastenzehen (Abb. 1a) sowie Papillomatosis cutis lymphostatica (Abb. 1b).

**Table 3:** 

Stadium 0	Kein klinisch apparentes Lymphödem. Es können aber schon pathologische Befunde im Lymphszintigramm sichtbar sein
Stadium I	Ödem von weicher Konsistenz, das nach Hochlagerung reversibel ist
Stadium II	Ödem mit sekundären Gewebeveränderungen. Das Ödem persistiert trotz Hochlagerung
Stadium III	Deformierende harte persistierende Schwellung, oft mit typischen Hautveränderungen

### Lipödeme

**Figure 2:**
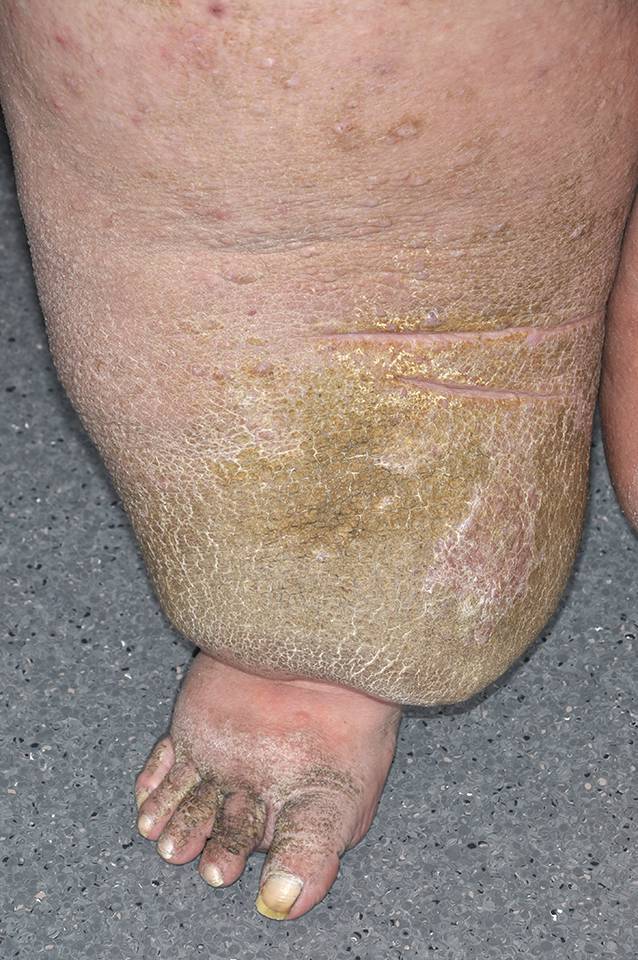

Das Lipödem sieht zwar wie ein Ödem aus, ist aber primär kein Ödem, sondern eine Fettgewebsvermehrung. Lipödeme beginnen meist in der Pubertät, Schwangerschaft oder Menopause und betreffen nahezu ausschließlich Frauen. Auch die Einteilung der Lipödeme wird in einer AWMF-Leitlinie [1] beschrieben (Tab. 4). Die Ätiologie ist weiterhin unbekannt; es kommt zu einer Hypertrophie und Hyperplasie der Fettzellen. Typischerweise sind beide Beine und/oder Arme symmetrisch von der Umfangsvermehrung betroffen; die Füße und Hände sind ausgespart (Abb. 2). Charakteristisch ist zudem die Druckschmerzhaftigkeit, die oft auch mit Spontanschmerzen einhergeht. Durch eine erhöhte Kapillarfragilität kommt es zu einer Hämatomneigung [15]. Im Verlauf der Erkrankung kann zudem selten ein sekundäres Lymphödem auftreten, das meist durch eine begleitende Adipositas bedingt ist und die exakte klinische Zuordnung erschwert. Von der Adipositas unterscheidet sich das Lipödem durch die dysproportionale Fettgewebsvermehrung („voluminöse Beine bei schlankem Oberkörper“) und die obligatorischen Schmerzen der voluminösen Extremitäten.

**Table 4:** 

Stadium 1	Glatte Hautoberfläche mit gleichmäßig verdickter, homogen imponierender Subkutis
Stadium 2	Unebene, überwiegend wellenartige Hautoberfläche, knotenartige Strukturen im verdickten Subkutanbereich
Stadium 3	Ausgeprägte Umfangsvermehrung mit überhängenden Gewebeanteilen, die auch als Wammen bezeichnet werden

### Stemmer-Zeichen

**Figure 3:**
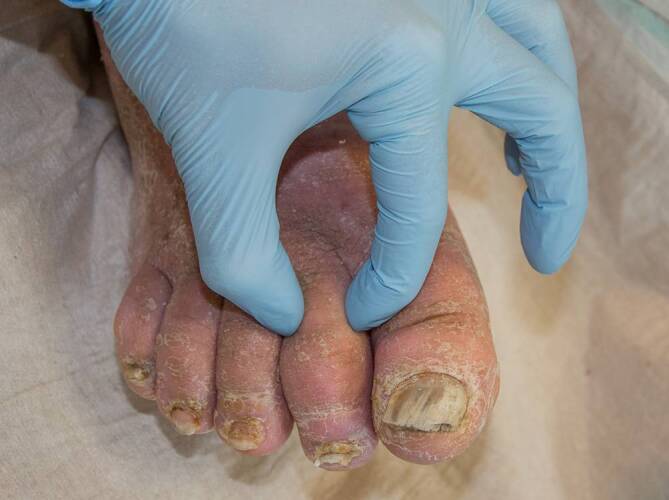

Die Testung des Stemmer-Zeichens ist eine einfach und schnell durchzuführende palpatorische Methode für die Diagnostik von Lymphödemen [24]. Hierbei wird die Haut auf der Oberseite der Grundphalanx der zweiten oder dritten Zehe mit 2 Fingern angehoben. Kann keine Hautfalte angehoben werden bzw. ist die Haut nicht mehr eindrückbar, ist das Stemmer-Zeichen positiv (Abb. 3). Lässt sich eine Hautfalte anheben, ist das Stemmer-Zeichen negativ (Tab. 5). Ein positives Stemmer-Zeichen weist auf das Vorliegen eines Lymphödems hin. Allerdings schließt ein negatives Stemmer-Zeichen ein Lymphödem nicht sicher aus [10]. Insbesondere Betroffene mit sekundären Beinlymphödemen, z. B. nach Lymphknotenoperationen im Bauchraum oder in der Leiste, haben oft ein negatives Stemmer-Zeichen. Etwa 15 % der erwachsenen Bevölkerung in Deutschland weisen ein positives Stemmer-Zeichen auf [17]. Diese Prävalenz steigt mit zunehmendem Lebensalter an.

**Table 5:** 

Grad 0	Normale Hautfalte
Grad 1	Verdickung der Hautfalte um 0,5–1 cm
Grad 2	Verdickung der Hautfalte um > 1 cm
Grad 3	Verdickung der Hautfalte um > 1 cm und zusätzlich Induration und/oder Papillomatosis cutis lymphostatica

### Godet-Zeichen

**Figure 4:**
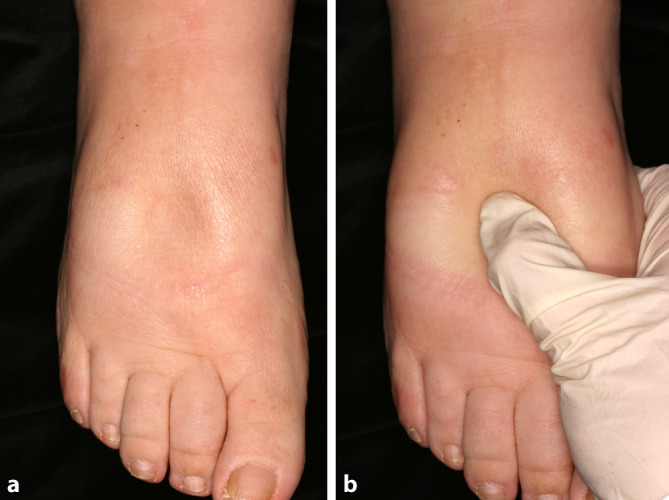

Das Godet-Zeichen wird im englischsprachigen Raum auch als „pitting edema“ bezeichnet. Hierbei wird auf das zu untersuchende Gewebe beispielsweise mit dem Daumen bei der Palpation für mindestens 10 s Druck ausgeübt (Abb. 4a). Dadurch entsteht eine Eindellung, die sich entweder sofort oder nach einer bestimmten Erholungszeit wieder ausgleicht. Es finden sich in der Literatur sehr unterschiedliche Angaben zu der Art und der Dauer, der Durchführung sowie zu den resultierenden Ergebnisse des Godet-Zeichens (Tab. 6; [25]). Die hier angegebenen Werte für Tiefe und Erholungszeit sind daher lediglich als ungefähre Richtwerte zu verstehen, da die Durchführung der Drucktestung hinsichtlich Zeit und Anpressdruck nicht gut zu standardisieren ist. Venöse Stauungsödeme sind ebenso wie andere hypoproteinämische Ödeme eindrückbar und hinterlassen somit bei der Testung des Godet-Zeichens sichtbare Dellen im Gewebe (Godet-Zeichen positiv) (Abb. 4b). Lymphödeme sind hingegen meist wegen des hohen Proteingehalts der Ödemflüssigkeit nicht eindrückbar (Godet-Zeichen negativ).

**Table 6:** 

Grad	Tiefe (mm)	Erholungszeit (s)
0	0	Keine
1	2	Unmittelbar
2	4	< 15
3	6	15–30
4	8	> 30

## Beeinflussung des Ausmaßes des Ödems

Die Ausprägung und das Ausmaß des Ödems basieren auf einer Grunderkrankung, wie beispielsweise einer Lymphabflussstörung oder einer Veneninsuffizienz [22]. Trotz vergleichbarer und stabiler Grunderkrankung kommt es zu unterschiedlicher Ausprägung der Ödeme, die von verschiedenen zusätzlichen Faktoren abhängig sind (Tab. 7).

**Table 7:** 

Position des Patienten in Ruhe vor der Untersuchung
Ausmaß der Bewegung des Patienten vor der Untersuchung
Position des Patienten während der Untersuchung, z. B. Liegen, Stehen, Sitzen
Tageszeit, z. B. abends stärkere Ödemausbildung als morgens
Kompressionstherapie oder keine Kompressionstherapie vor der Untersuchung
Untersuchungstechnik der Ödemquantifizierung
Untersucherabhängige Faktoren
Kardiovaskuläre Komorbidität oder Co-Medikation
Änderungen in der Co-Medikation mit Volumeneffekten
Behandlung einer Varikose

Wenn Patienten sich aus der Rückenlage kommend hinstellen, steigt der hydrostatische Druck in den Venen an. Es kommt zu einer Venenerweiterung und zu einer unmittelbaren Volumenzunahme um etwa 500 ml [23]. Bei einem Positionswechsel vom Sitzen zum Stehen steigt das Venenvolumen um etwa 190 ml. In perimetrischen Messungen konnte gezeigt werden, dass das Beinvolumen beim Wechsel vom Liegen zum Stehen um etwa 2,5 % ansteigt [18]. Bei gesunden Freiwilligen steigt das Beinvolumen um 51 ± 32 ml innerhalb von 30 min Stehen an [16]. Dies spiegelt sich auch in wiederholten volumetrischen Messungen wider, die über 15–30 min wiederholt wurden. Hier kam es während der wiederholten Messungen zu zunehmend größeren Werten mit einer Zunahme um 50 ml, wenn die Patienten im Stehen untersucht werden [23]. Abgesehen von den orthostatisch bedingten zirkadianen Änderungen der Beinvolumina gibt es eine Reihe weiterer Faktoren, die tageszeitliche Schwankungen der Ödeme verursachen.

Bei der Beurteilung des Ausmaßes des Ödems sollten kardiale Komorbiditäten und Medikationen berücksichtigt werden. Auch kardiale Ödeme unterliegen großen Schwankungen im Tagesverlauf, was gerade bei den oft älteren Wundpatienten eine relevante Rolle spielt. Daher sollten bei veränderten Ausprägungen des Ödems internistische Symptome und insbesondere auch Änderungen der Medikation, wie beispielsweise Diuretika, in Betracht gezogen werden. Neben diesen positions- und zeitabhängigen Änderungen des Ödemvolumens spielt auch die Messgenauigkeit des jeweiligen Untersuchungsverfahrens eine große Rolle.

## Messtechniken zur Ödemkontrolle

Das klinisch nachvollziehbare Abschwellen des Ödems gibt Aufschluss über die Effizienz der therapeutischen Maßnahmen. Eine regelmäßige Kontrolle der Ödemreduktion bestätigt somit den Therapieerfolg oder weist auf notwendige Anpassungen in der Therapie hin.

### Umfangsmessungen

**Figure 5:**
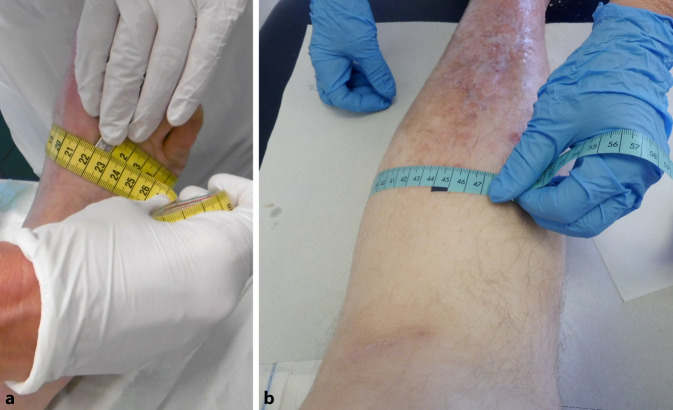

**Figure 6:**
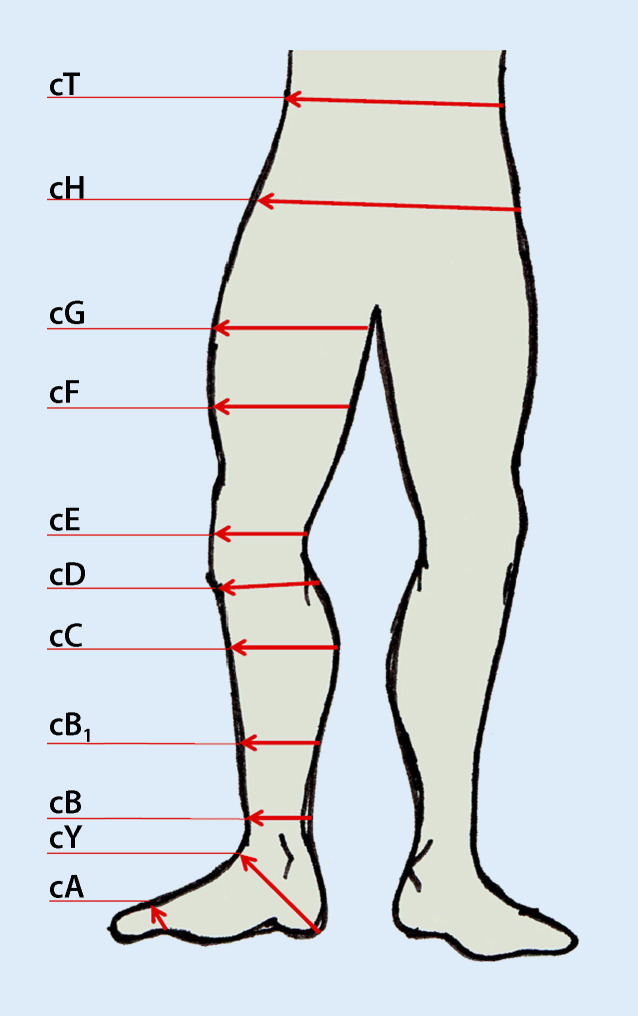

Es empfiehlt sich im Zusammenhang mit einem regelmäßigen Monitoring, beispielsweise wöchentlich, Vorfuß- (Abb. 5a), Knöchel- und Wadenumfang (Abb. 5b) zu messen [6]. Diese Messungen sollten immer an derselben Lokalisation erfolgen, wodurch der Erfolg von entstauenden Maßnahmen ersichtlich wird. Ist allerdings innerhalb 1 Woche keine Umfangminderung festzustellen, kann dies u. a. an einer ineffizienten Kompressionstherapie, aber auch an einer nicht vorhandenen bzw. nur sehr eingeschränkten Funktion der Venenpumpen liegen oder daran, dass der Patient die Kompressionsversorgung nicht bzw. nicht adäquat trägt [9]. Um die Vergleichbarkeit der Ergebnisse der Umfangmessung zu gewährleisten, können die Messpunkte am Vorfuß, am Knöchel und an der Wade mit einem Hautmarkerstift gekennzeichnet werden. Eine Orientierung bietet die Standardmethode zur Vermessung des Beines für die Versorgung mit medizinischen Kompressionsstrümpfen (MKS), die Messpunkte an Vorfuß (A), Knöchel (B) und Wade (C) definiert (Abb. 6). Die Umfangerfassung wird händisch mit einem Maßband direkt auf der Haut durchgeführt. Aus hygienischen Gründen sind Einmalmaßbänder aus Papier, beispielsweise erhältlich bei Strumpfherstellern, oder wischdesinfizierbare Maßbänder aus Kunststoff zu nutzen. Die Vermessung sollte immer unter gleichen Bedingungen stattfinden und im entstauten Zustand möglichst morgens vor dem Aufstehen oder direkt nach Abnahme der Kompressionsbandagierung vor Anlage einer neuen Versorgung erfolgen. Wenn es dem Betroffenen oder seinen Angehörigen möglich ist, dies selbstständig durchzuführen, können die Ergebnisse in einer Art „Entstauungstagebuch“ festgehalten werden. Dies fördert das gesundheitsbezogene Selbstmanagement, das Empowerment und die Adhärenz der Betroffenen. Über die Fachgesellschaft Initiative Chronische Wunden (ICW) e. V. ist ein solches „Bewegungstagebuch – Übungen zur Ödemreduktion“ erhältlich. Dies soll Betroffene anhand von bebilderten Beispielen zu täglichen Bewegungsübungen anregen und zu 1‑mal wöchentlichen Umfangmessungen motivieren, die dann anschließend im Tagebuch notiert werden. Zudem ist es ein hilfreiches Kommunikationsmittel für den Austausch mit den Versorgern.

### Sonographie

**Figure 7:**
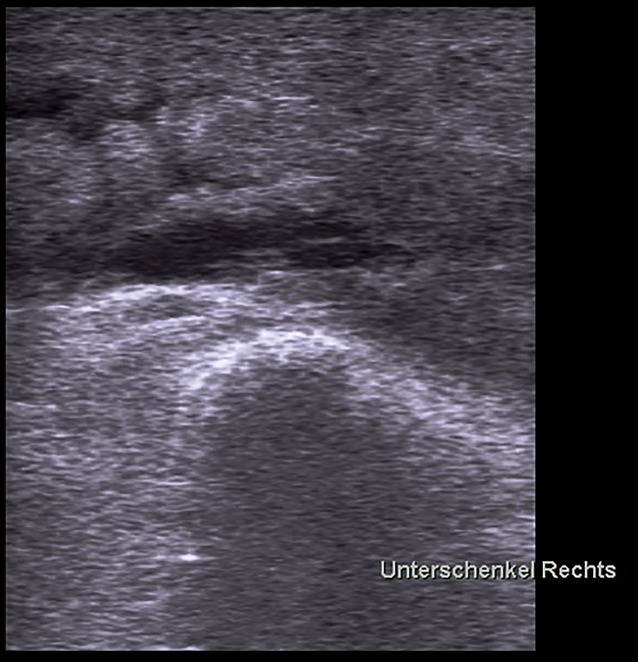

Da Ödeme meist in der Subkutis lokalisiert sind, stellen sie sich dort als echoarme Spalträume dar, die jedoch nicht den lymphatischen Strukturen zugeordnet werden können (Abb. 7). Die Stärke der Sonographie liegt in einer Visualisierung und Objektivierung von Ödemen im Unterhautfettgewebe [5]. In der Sprechstunde kann dies sinnvoll sein, um einerseits den Patienten anhand der Befunde zu verdeutlichen, dass das vorhandene Ödem noch nicht ausreichend behandelt ist. Umgekehrt kann aber auch sonographisch gezeigt werden, dass keine Ödeme nachweisbar sind. Dies kann beispielsweise ein Zeichen dafür sein, dass in dieser Situation eine manuelle Lymphdrainage nicht mehr erforderlich ist, sondern die bereits durchgeführte Kompressionstherapie offenbar ausreichend zu einer Ödembeseitigung geführt hat. Eine Differenzierung unterschiedlicher Ödemtypen mittels Sonographie ist nicht mit ausreichender Trennschärfe möglich [5]. Daher ist die Sonographie derzeit lediglich zum qualitativen Nachweis von Ödemen geeignet.

### Messtechniken zur Volumenmessung

Das Volumen der Beine kann mit verschiedenen Techniken gemessen werden. Relativ einfach sind die Messung der Beinumfänge an mehreren Punkten und die Bestimmung der Abstände zwischen diesen Umfängen, woraus in mathematischen Modellen das Volumen berechnet werden kann [20]. Bei der Volumenberechnung nach Kuhnke wird der Umfang der Extremität alle 4 cm gemessen. Da man hier von einer zylindrischen Form der Extremität ausgeht, ist die Berechnung nicht sehr exakt [11]. Aufwendiger sind optoelektronische Messverfahren wie die Perometrie, bei der ein Metallrahmen mit 2 Reihen von 240 bis 300 Infrarotfototransistoren am Bein entlanggeführt wird und über Mikroprozessoren Umfänge in mm am Bein gemessen werden, woraus dann das Volumen berechnet werden kann [20]. Aktuellere Messtechnik sind dreidimensionale Volumenmessgeräte wie das Bodytronic 600 (Bauerfeind, Zeulenroda), bei dem ein Lichtgitter auf Fuß, Unterschenkel und Oberschenkel gestrahlt wird, um ein dreidimensionales Modell dieser Strukturen zu berechnen. Die Messung erfolgt innerhalb von 50 s, sodass keine Volumenverschiebungen durch die Messdauer an sich zu erwarten sind. Bei dem Vergleich dieser optischen Volumenmessungen mit einer Computertomographie zeigt sich eine gute Genauigkeit und Reliabilität des Verfahrens in der Bestimmung von Bein- und Fußumfängen [13]. Eine weitere neue Option ist die dreidimensionale Messung der unteren Extremitäten mittels Laserscanners. Die sehr genauen und gut reproduzierbaren Ergebnisse, die mit dieser Methode erzielt werden können, wurden aktuell in einer prospektiven klinischen Studie mit 30 Patienten mit chronisch venöser Insuffizienz (CVI) bestätigt [26].

### Wasserplethysmographie

Die Wasserplethysmographie gilt weiterhin als Goldstandard für Volumenmessungen des Unterschenkels und Fußes. Allerdings muss das Verfahren unter standardisierten Bedingungen mit möglichst gleicher Tageszeit der Messung, mit gleichen Ruhebedingungen der Patienten und technisch perfekter Durchführung ausgeführt werden, um wirklich die Stärken der Methode nutzen zu können. Durch Anwendungsfehler der Methode kann es zu messtechnisch bedingten Volumenunterschieden von über 300 ml kommen [23].

## Diskussion und praktische Empfehlungen

Die Diagnostik und Therapie von Ödemen der Beine ist gerade im Sektor der Wundbehandlung sehr wichtig und wird von Patienten und Therapeuten oft unterschätzt. Die Ausprägung von Ödemen wird von einer Vielzahl von Faktoren beeinflusst. Daher kann eine quantitative Ödemkontrolle im klinischen Alltag problematisch sein. Die Quantifizierung von Veränderungen von mäßiggradig ausgeprägten Ödemen, die in einer Größenordnung der normalen tageszeitlichen Schwankungen von etwa 80 ml pro Unterschenkel liegen, sind im klinischen Alltag nicht hinreichend genau zu bestimmen, sondern erfordern sehr ausgefeilte Messtechniken, die nur unter Studienbedingungen gewährleistet werden können.

Für die Praxis wird empfohlen, zunächst zu prüfen, ob überhaupt ein Ödem vorliegt. Ist ein Ödem weder klinisch noch sonographisch nachweisbar, sind keine weiteren Volumen- oder Umfangsmessungen erforderlich. Ist klinisch oder sonographisch ein Ödem nachweisbar, sollte entschieden werden, ob ein stark ausgeprägtes Ödem mit einem Godet-Zeichen > Grad 1 vorliegt. In dieser Situation sind in der klinischen Routine Umfangsmessungen, wie zuvor beschrieben, sinnvoll, so lange bis eine zufriedenstellende Entstauung bis zu einem fehlenden oder geringgradigen Ödem erreicht worden ist. Therapeutisch stehen dann nach Ausschluss der entsprechenden Kontraindikationen [8] verschiedene Arten der medizinischen Kompressionstherapien [3], intermittierenden Kompression (IPK) [4] und/oder Lymphdrainage im Rahmen einer komplexen physikalischen Entstauungstherapie (KPE) [2, 21] zur Verfügung.

## Fazit für die Praxis

- Massiv ausgeprägte Ödeme mit ausgeprägter Umfangs- und Volumenzunahme schränken die Lebensqualität der Betroffenen stark ein.
- Ödeme können unterschiedliche Ursachen und klinische Ausprägungen haben.
- Regelmäßige Messung und Dokumentation der Ödeme zeigen, ob therapeutische Maßnahmen erfolgreich sind.
- Einfache klinische Testungen von Ödemen sind beispielsweise Stemmer- und Godet-Zeichen.
- Klinische Untersuchungen sind in Kombination mit Sonographie gut geeignet und wenig aufwendig, um Ödeme zu beurteilen.
